# The relevance of knowledge, perception, and factors that influence contraceptive use among married women living in Uaddara Barracks, Ghana

**DOI:** 10.3389/fgwh.2023.1110024

**Published:** 2023-08-15

**Authors:** Daisy Afra Lumor, Christian Obirikorang, Emmanuel Acheampong, Yaa Obirikorang, Hubert Owusu, Sam Newton

**Affiliations:** ^1^ Department of Midwifery, St. Patrick's Nursing and Midwifery Training School, Offinso, Ghana; ^2^ Department of Molecular Medicine, School of Medicine and Dentistry, Kwame Nkrumah University of Science and Technology, Kumasi, Ghana; ^3^ Department of Genetic and Genome Biology, University of Leicester, Leicester, United Kingdom; ^4^ Centre for Precision Health, School of Medical and Health Sciences, Edith Cowan University, Joondalup, WA, Australia; ^5^ Department of Nursing, Garden City University College, Kumasi, Ghana; ^6^ Department of Global and International Health, School of Public Health, Kwame Nkrumah University of Science and Technology, Kumasi, Ghana

**Keywords:** contraceptive use, condom, women, Ghana, Africa

## Abstract

**Background:**

Contraceptive use has many advantages for personal growth and societal advancement, but there is still the problem of unmet needs for women, which highlights the gap between women's reproductive intentions and contraceptive use. This study investigated knowledge, perceptions, and factors that influence contraceptive use among married women living in a military base in Ghana.

**Methods:**

This cross-sectional study involved 350 married women between the ages of 20 and 58 years at the Uaddara Barracks, Kumasi. A structured questionnaire was used to collect information on the background, knowledge, perceptions on contraceptive use, and contraceptive methods used by participants. Data was entered into an Excel sheet and analysed using R version 4.2.1.

**Results:**

Most of the participants were between the age range of 36 and 40 years (25.5%). Almost all study participants (97.4%), had heard about contraceptives with 80.6% showing a high level of knowledge on contraceptives. The majority of the women (84.6%) had previously used some form of contraceptives and 53.1% presently do. More than half of the participants (69.4%) had a positive perception of contraceptive use; 80.6% responded it was their own decision to use contraceptives, and 80.3% had the support of their husbands. Husbands' support of contraception resulted in a 5 times higher usage of contraceptives among women (aOR =  5.35; *p* < 0.001) while women who were married to military men were 45% (aOR = 0.45; *p* = 0.007) less likely to use contraceptive when compared to civilian wives. Demographic characteristics like being above 40 years (aOR = 0.25; *p* = 0.014), being a housewife (aOR = 0.42; *p* = 0.043) and working in the private sector (aOR = 0.33; *p* = 0.015) were significantly linked with less contraceptive use.

**Conclusion:**

The study showed that women used contraceptives at a rate that was much higher than the national norm at the Uaddara Barracks, demonstrating the beneficial influence men had on women's contraceptive use. This thereby underscores the need for interventional policies that prioritized the male as much as women, while emphasizing the benefits of contraceptive use to the family and not just as an awareness program only.

## 1. Introduction

The global population is increasing more rapidly than the Earth can handle (1). The world's population is projected to reach 8.5 billion in 2030, 9.7 billion in 2050, and 11.2 billion by 2,100 (2). Much of this population growth is projected to be in the low-middle-income countries in Sub-Saharan Africa (3). Ghana's population increased by 30.4 percent from 18,912,079 in 2,000 to 24,658,823 in 2010 (4). This increase places an enormous burden on the country's ability to adequately provide for its citizens by putting a strain on essential social services such as education, health, water, and housing (1, 5). Thus, the promotion of family planning services such as modern conceptive use as a means of cutting down fertility rates, and empowering women in their childbearing age (3, 6). Not only that, family planning has enabled couples to choose the number and timing of childbirth while also serving to prevent unwanted pregnancies, and unsafe abortions, offering protection against sexually transmitted diseases such as HIV/AIDS through such methods as condom use (7, 8). In addition, family planning is widely regarded as an important way to achieve Millennium Development Goals (MGDs) 4 and 5 through the reduction of maternal and child mortality (8).

However, while the global use of family planning services was estimated to be 57.4% in 2015, it has rather remained low in many Sub-Saharan countries such as Ghana (7, 9, 10). Over the years, the prevalence of modern contraceptive use among married women in Africa rose from 23.9% in 2012 to 28.5% in 2017 with the West African subregion recording a low rate of 20.0% that same year (11). In other parts of the world, the prevalence of contraceptive use rose from 60.9% to 61.8% in Asia, while remaining steady at 66.7% in Latin America and the Caribbean (9, 12). Ghana's prevalence of contraceptive use stands at 27.1% with the country having missed its goal of reaching a 50.0% target rate in 2020 (11, 13).

It is estimated that about 225 million women in developing countries would like to delay or stop childbearing but are not using any contraceptive method, which is an unmet need for family planning (14). Women with an unmet need are those who are fecund and sexually active but are not using any method of contraception while reporting not wanting any more children or expressing a desire to delay the next child (14). The concept of unmet need points to the gap between women's reproductive intentions and their contraceptive behavior (15). Several factors have been associated with the low uptake of contraceptives. Some studies in Ghana have shown that knowledge of contraceptives, their availability, as well as easy access to contraception—particularly among young people, poorer segments of populations, or unmarried people—all have a positive influence on their use (6, 16, 17). Other factors include a limited choice of methods; fear of or experience of side effects; cultural or religious opposition; poor quality of available services; user and provider bias; and gender-based barriers (18–20).

The various factors that influence the use of modern contraceptive methods have been noted to differ across countries (16). In Ghana, both government and non-governmental organizations have employed various campaign programs to improve the use of modern contraceptives use across the country. While this has gained some success, specifically in improving awareness and knowledge of contraceptive methods across much of the country, women with unmet needs remain high (21). However, many studies exploring modern contraceptive use within the country have rather focused on the general population (6). This makes the current study important as it explores the factors that influence the use of modern contraceptive methods among women within the barracks, a place where military deployments routinely separate families over a long period of time. The information gathered would go a long way in formulating long-term campaign programs on how to effectively reach our military women and the wives of our military men with such unmet needs.

## 2. Materials and method

### 2.1. Study design and setting

This was a quantitative cross-sectional study conducted among 350 married women living in the barracks at the 4BN Uaddara Barracks, Kumasi. The barracks is about 10 min drive away from the city center, right beside the Komfo Anokye Teaching Hospital. It houses about 2,400 individuals which includes military personnel and their spouses, as well as civilian employees and their spouses who receive reproductive and other health services from the teaching hospital, nearby private clinics, and midwife-managed maternity homes.

### 2.2. Data collection

A structured questionnaire was used to obtain information from all study participants. The structured questionnaire was developed based on a review of published articles (22, 23) It was made up of four sections. Section A collected information on the sociodemographic characteristics of respondents. Section B was designed to collect information about contraceptive use. Section C constituted information on perceptions towards contraceptive methods. Section D consisted of questions about the involvement of their partners in contraceptive use. The study was pretested with 20 women of similar socio-cultural characteristics. Weaknesses identified during the pre-test were rectified before the final administration of the questionnaire. The independent variable of the study was contraceptive use among married women and the dependent variables of the study were knowledge, perceptions, compliance, and factors influencing contraceptive use. These are defined as a married woman's awareness and information on modern contraceptive methods, attitudes, adherence to modern contraceptive methods, and influencing factors of modern contraception uptake. To assess these, questions were asked about awareness of modern contraceptive methods; ever-used contraceptive methods (condoms, oral contraceptives, withdrawal, Intrauterine device, several methods, or abstinence); reasons for uptake or withdrawal, or the urge to continue usage.

### 2.3. Data handling and management

The knowledge of respondents about contraception scored a point for a correct answer and zero for a wrong answer. After adding the scores median split was used to define the respondents having low knowledge (those who were below the median) and high knowledge (those above the median mark). Similarly, for perception, using the point Likert scale, with agreed scoring one point and disagreed, and I don't know scoring zero for correctly answered questions, and vice versa for incorrectly answered questions. Scoring for attitude was done using a median split into dichotomous data; scores that are up to or more than the mean was regarded as a positive attitude and those below the median had a negative attitude.

### 2.4. Data analysis

Data was entered in an Excel spreadsheet for Windows and analyzed using R version 4.2.1. Data for continuous variables between two groups were presented as means ± SD standard deviation. Categorical variables were presented as frequency (*n*) and percentage (%). Women's contraceptive use were regressed on socio-demographic variables, knowledge level, perception of contraceptive use, partner's demographics, partner's support and use of the male condom, as well as on factors relating to marriage, fertility, and desire for more children. Significance was defined as a *p*-value of <0.05.

### 2.5. Ethical consideration

Ethical clearance was sought from the Committee on Human Research, Publications, and Ethics (CHRPE) (reference: CHRPE/AP/384/18) of KNUST and the Commanding Officer of the 4th Infantry Battalion, Uaddara Barracks, Kumasi. All respondents signed a written consent form, they were informed that taking part in the research was voluntary and that they can opt out at any time.

## 3. Results

### 3.1. Demographic characteristics of the study participants and their partners

Table 1 summarizes the socio-demographic characteristics of participants. The study involved 350 participants between the ages of 20 and 58 years, many of whom (25.5%) were within the 36–40 years bracket. The majority of participants (98.3%) have had secondary education, 83.4% were Christians, 40.9% were government employees, and almost half (49.7%) got married between the ages of 23 and 27 years. Most of the participants responded that their husbands got married between the ages of 28–32 years (43.4%), and 60.3% said their husbands have had an education to the tertiary level. A higher proportion of participants responded that their husbands were military personnel (77.4%) (Table 1).

**Table 1:** Demographic characteristics of study participants.

Variable	Frequency (*n* = 350)	Percentage (%)
Age bracket (years)		
<25	53	15.1
26–30	88	25.1
31–35	84	24.0
36–40	89	25.5
≥41	36	10.3
Educational level		
No formal education	6	1.7
Basic	57	16.3
Secondary	149	42.6
Tertiary	138	39.4
Religion		
Traditionalist	6	1.8
Christian	292	83.4
Muslim	52	14.9
Occupation		
Government employee	148	40.9
Housewife	47	13.7
Private employee	35	10.2
Self-employed	120	35.1
Age at marriage (years)		
<18	23	6.6
18–22	77	22.0
23–27	174	49.7
28–32	66	18.9
>32	10	2.9
Husbands’ age at the time of marriage		
Don’t know	25	7.1
18–22	15	4.3
23–27	84	24.0
28–32	152	43.4
>32	74	21.1
Husband's educational level		
Basic	6	1.7
Secondary	133	38.0
Tertiary	211	60.3
Husband's occupation		
Military personnel	271	77.4
Civilian employee	79	22.6

Values are presented as proportions of participants that gave response to the variables presented.

### 3.2. Contraceptive use among the participants

**Figure 1:**
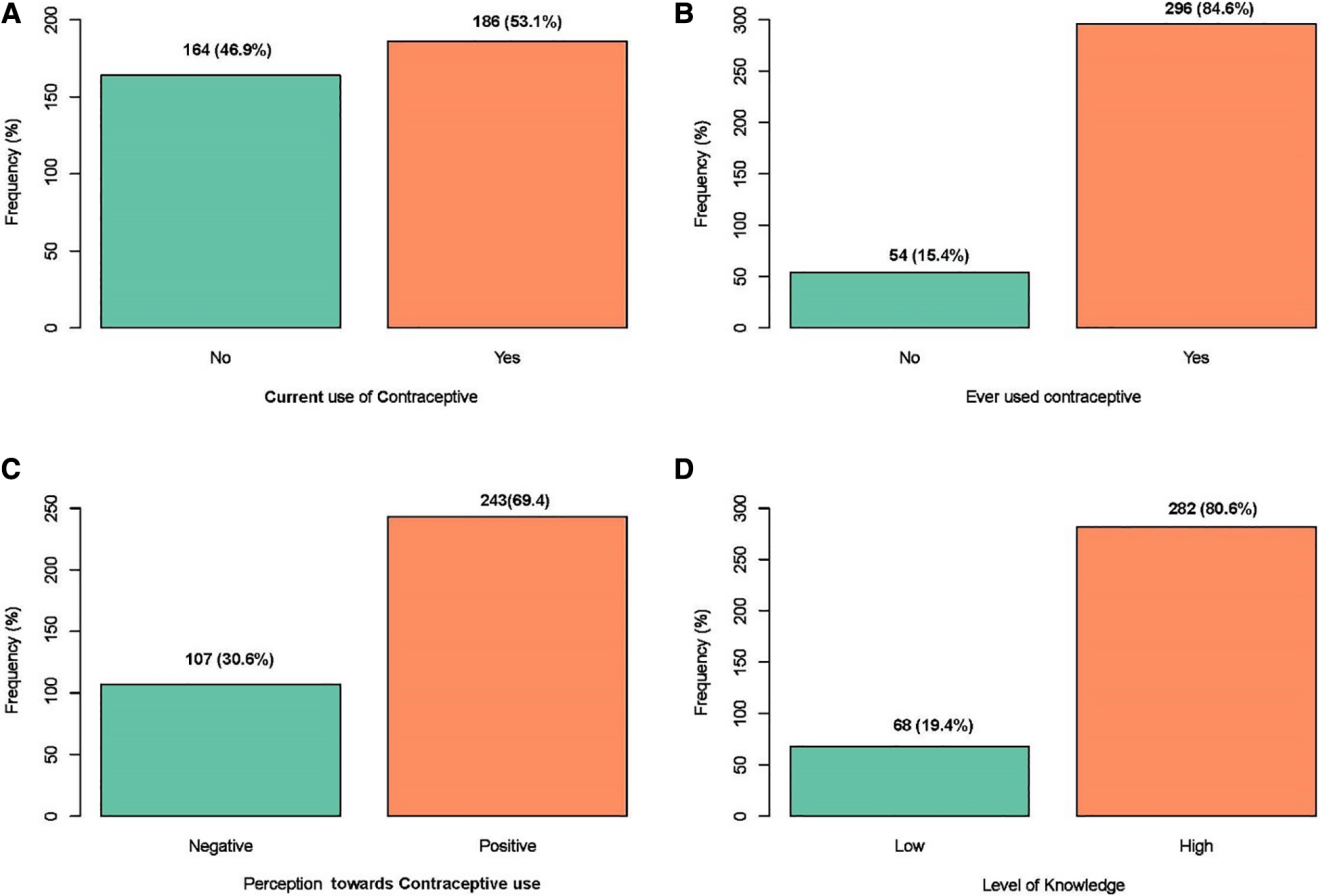
A graph showing the frequency distribution of women's: (**A**) current contraceptive use; (**B**) if they have ever used any contraceptive method; (**C**) their perception toward contraceptive use; (**D**) their level of knowledge on contraception.

Most of the participants (84.6%) have previously used some form of contraceptives, while 53.1% were currently doing so (Figures 1A,B). Injectable was the most used current contraceptive method (26.7%) (Supplementary Table S1). The majority of the participants (80.6%) indicated that they chose to use contraception on their own and 51.1% mentioned they had never had any side effects. In addition, 80.3% responded that their husbands supported them in using contraception, and 67.9% mentioned their husbands used the male condom respectively (Supplementary Table S1).

### 3.3. Awareness and knowledge of contraceptive use among participants

Almost all study participants (97.4%), have heard about contraceptives and the principal source of information was the media/internet (64.5%) (Supplementary Table S2). Condoms (74.5%) were the most widely known contraceptive method. More than half (52.5%) of the participants knew withdrawal as a traditional contraceptive method. A major proportion of the participants (64.5%) knew about the sources of contraceptives with the family planning unit being the most frequently represented source (56.4%) (Supplementary Table S2). Overall, 80.6% of participants demonstrated a high level of knowledge of contraceptives (Figure 1D).

### 3.4. Perception of contraceptive use among participants

As shown in Supplementary Table S3, a majority of participants disagree with the fact that modern contraceptive services and commodities are inaccessible (69.7%), it is not easy to discuss sexual issues with a partner (62.0%), contraceptives are for females only (86.3%), and it is wrong to use contraceptives (82.9%) (Supplementary Table S3). On the other hand, more than half of the study participants agreed that contraceptives were acceptable at the barracks (63.7%) and that couple counselling could improve male involvement in contraceptive use (70.9%) (Supplementary Table S3). Altogether, 69.4% of participants showed positive perceptions towards contraceptive use (Figure 1C).

### 3.5. Married life of study participants

Most of the participants (40.3%) have been married for 1–5 years, and 45% of them had 1–2 children. One hundred and sixty-seven of the participants (47.7%) desired a male child and 40.9% desired a female child. More than half (69.1%) of the study subjects anticipated having 3–4 children while only 5.1% projected to have 7–8 children. Higher proportions of the participants had no problems taking decisions in marriage (61.1%) and the remaining 38.9% noted to have problems taking decisions in marriage (Supplementary Table S4).

### 3.6. Factors associated with contraceptive use among participants

**Figure 2:**
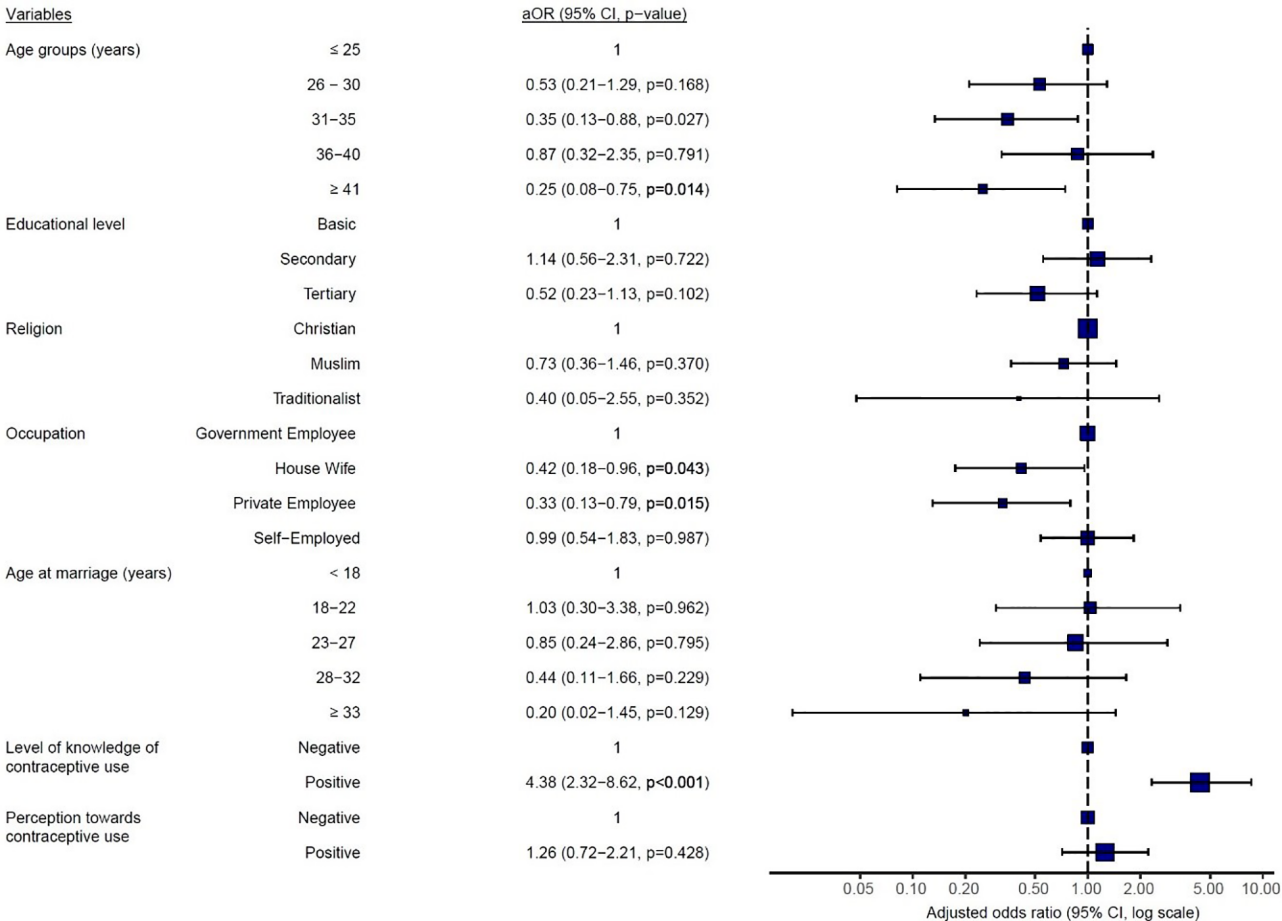
Multivariate logistic regression analysis of women's current contraceptive use on socio-demographic variables, knowledge level, and perception of contraceptive use. aOR, adjusted odds ratio, CI, confidence interval, *p* < 0.05 is statistically significant.

Multivariate logistic regression analysis showed that participants who were above 40 years were 25% less likely to use contraceptives when compared to women who were 25 years old or younger (aOR = 0.25; *p* = 0.014), where women who were housewives (aOR = 0.42; *p* = 0.043) or those who worked in the private sector (aOR = 0.13; *p* = 0.015) were associated with a 25% and 42% less likelihood to use any form of contraceptives when compared, and those who were government employees (Figure 2). No significant relationship was observed between participants likelihood to use contraceptives and their educational level, religion, and age of marriage. Nonetheless, participants with a higher knowledge of contraceptives were 4 times more likely to use them than those who did not have much knowledge on contraceptives (OR = 4.38; *p* < 0.001).

**Figure 3:**
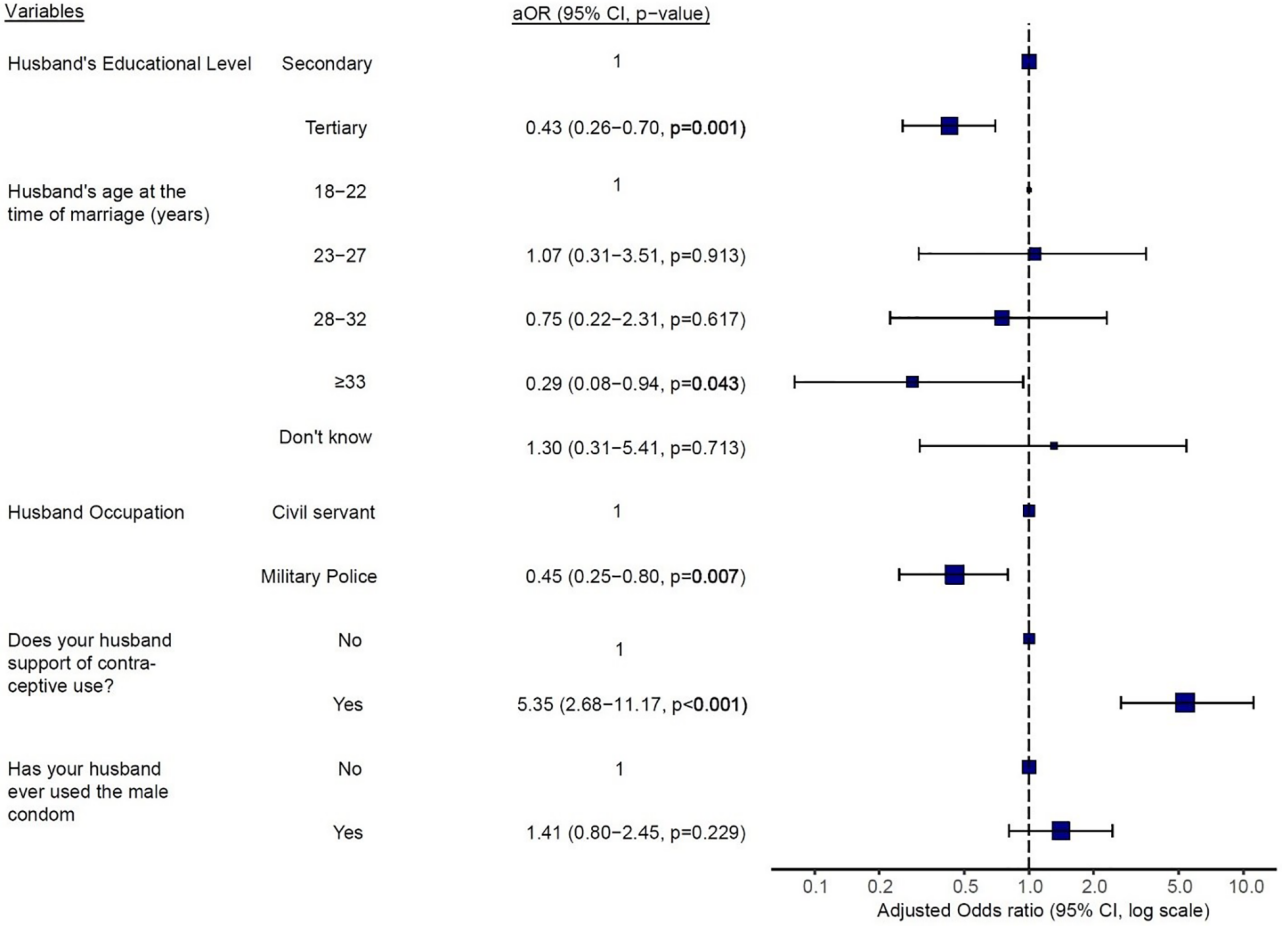
Multivariate logistic regression analysis of women's current contraceptive use on partner's demographics and partner's support and use of the male condom. aOR, adjusted odds ratio, CI, confidence interval, *p* < 0.05 is statistically significant.

As depicted in Figure 3, participants whose husbands were 33 years or older at the time of marriage were significantly less likely to use contraceptives (aOR = 0.29; *p* = 0.043) when compared to those whose husbands got married between 18 and 22 years. Moreover, participants with husbands who have attained a tertiary level of education (aOR = 0.43; *p* = 0.001) or were married to Military Personnels (aOR = 0.45; *p* = 0.007) were at reduced odds of using contraceptives when compared to those whose husbands have only attained a secondary school level of education or were civil servants respectively (Figure 3). However, participants whose husbands support contraception were 5 times more likely to use contraceptives (aOR = 5.35; *p* < 0.001) than those whose husbands were not in support of contraceptive use (Figure 3).

**Figure 4:**
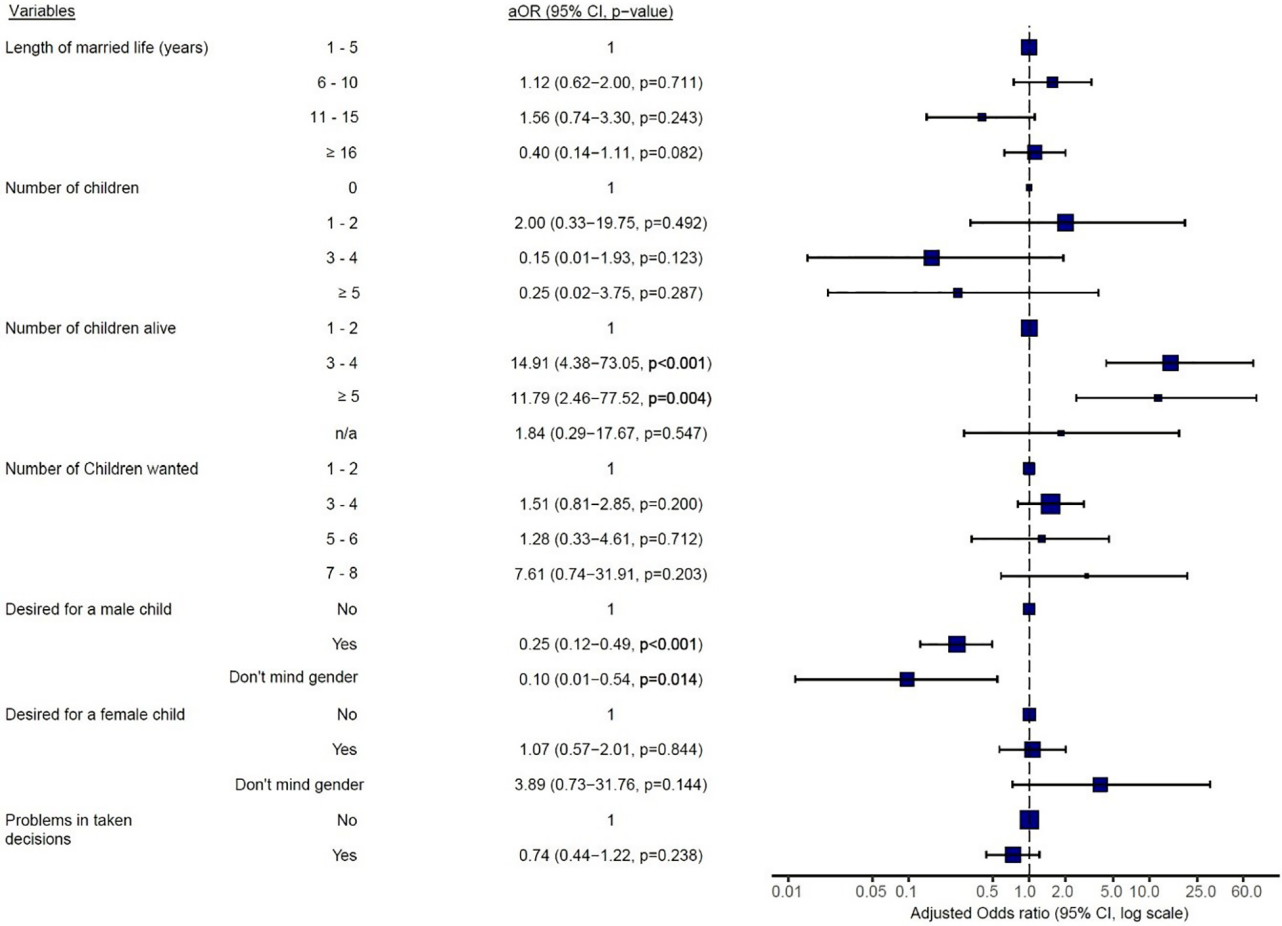
Multivariate logistic regression analysis of women's current contraceptive use on factors relating to marriage, fertility, and desire for more children. aOR, adjusted odds ratio, CI, confidence interval, *p* < 0.05 is statistically significant.

In addition, married women who had 3–4 children (aOR = 14.91; *p* < 0.0001) or ≥5 children alive (aOR = 11.79; *p* = 0.004) were 14 and 11 times respectively more likely to use contraceptives than women with 1–2 children alive (Figure 4). On the other hand, women who wanted male children were 25% less likely to currently use any form of contraceptives (aOR = 0.25; *p* < 0.0001) when compared to women who had no such desire. The length of participant's married life, desire for a female child, and participant's difficulties in taking decisions were all not significantly associated with their current use of contraceptives (Figure 4).

## 4. Discussion

Contraceptive use has many advantages, including, control of fertility and, as a result, population growth (21) and letting people decide how many and when to have children (24). There is an unmet need for women, despite prioritizing its acceptance (15). This study determined the knowledge, perceptions, and factors that influence contraceptive use among married women living in Uaddara Barracks, a military base in Kumasi, Ashanti Region.

In this study, a high degree of knowledge and awareness of contraceptives was observed among the study participants, which was consistent with the findings by Marrone et al, who reported that the Ghanaian population had a sufficient level of awareness level of contraceptive use (both modern and traditional) (21). Our results revealed that nearly all respondents could indicate at least one modern method of contraception, which is consistent with findings from other recent studies which showed a near-to-total universality of women's knowledge of contraceptive methods (6, 18, 25). The three modern contraceptives that the study participants most frequently reported using—both currently and in the past—were pills, injections, and condoms. Our results further reveal that injectables were the most used current contraceptive method. Similar findings have been reported by the Ghana Statistical Service (26). Aryeetey et al. (18), reported that estrogen/progesterone pills, male condoms, and injectables were the most commonly used contraceptive methods because they are the most frequently provided methods in many Ghanaian family planning centers. Nevertheless, due to wide media publicity in the fight against HIV/AIDS, many women were already familiar with condoms as a method of contraception (27). Moreover, other studies have also reported that a majority of reproductive-age women gravitate toward the use of injectables (23, 25). Eliason et al. reported that injectables are a preferred option, especially for new contraceptive users because certain communities associated the use of modern contraceptives with promiscuity. Similarly, Kamangu also notes that due to their high accuracy in pregnancy prevention and a reduction in the number of clinic visits for the service, injectables were a popular choice among Tanzanian women (25, 28).

In Ghana, modern contraceptive methods are available through the public health system as well as commercial outlets such as chemical stores, pharmacies, and other social marketing organizations (18). This was consistent with the present study where most women considered pharmacies and family planning units as their source for contraceptives. This reflects favorably on the national effort of making contraceptive methods more easily available and accessible. Nonetheless, participants’ ability to access contraceptive methods could also be a product of the urban setting in which they find themselves, which has been shown to provide more easily accessible avenues to obtain contraceptive methods than in rural areas (29).

We observed that a considerable proportion of the participants reported having ever used one or more contraceptive methods while more than half were currently still using contraceptives. These figures were much higher than the national contraceptive prevalence rate of 25% (26), or those of specific geographic areas within the country such as 36.9% in Ashaiman (6), or in other developing countries such as Nigeria (38%) (30), Pakistan (49%) (31), and India (45%) (32). The study also reported participants’ socio-demographic factors such as age, occupation, and age at marriage as factors that significantly influenced a person's decision to use modern contraceptives. These findings are consistent with those reported by Apanga and Adam in the Talensi District of Ghana (8) where age and occupation were associated with modern contraceptive use. Likewise, some other factors that influenced contraceptive use among married women in Uaddara Barracks included the number of children alive and a desire for a male child, with those in favor of having more than two children being less likely to use contraceptives. In addition, women above the age of 40 years reported a less likelihood to use contraceptives, which reflects a time when many would begin menopause. However, another fact that needs stressing is the role husbands play in their wives’ decision to use contraceptives.

Whereas 80.6% of married women currently using any form of contraceptive reported it was their own decision to use contraceptives, this was however possible with 80.3% of them having the support of their husbands. This was a far higher percentage rate than that observed in other studies in Ghana or Uganda where although a majority of women were aware of contraceptives, only a small percentage of them were able to access contraceptives due to opposition from their husbands (6, 8, 18). Thus, the reason for a higher rate of husband support observed in this study could be attributed to the occupational status and educational level of husbands, factors which were significantly associated with contraceptive use among women in this study. Again, this could also be a matter of rural-urban differences where rural residents are less likely to use contraceptive methods compared to urban residents (21). Nevertheless, women who were married to military men were also observed to be significantly less likely to use contraceptives when compared to women with civil employee husbands. This could reflect fact that wives whose military husbands are always away on deployment could be disincentivized or apathetic to the use of contraceptives when compared to civilian wives whose husbands are mostly present with them at home. In the same vein, this study also observed that participants whose husbands were above 32 years at the time of marriage were less likely to support the use of contraceptives, which could reflect a greater desire to give birth at that age.

Although the findings of this study are consistent with other findings, the cross-sectional methodology and small sample size utilized in this present study made it difficult to determine the causal effect of the relationship between demographic characteristics, knowledge and marital factors, and contraceptive usage.

## 5. Conclusion

A large majority of married women at the Uaddara Barracks were adequately informed about contraceptives, with a higher percentage having ever used and continuing to use them than the national average. Women married to military men were found to be less likely to use contraceptives when compared to spouses of civilian employees living in the barracks. Likewise, the support of husbands for their wives' usage of contraceptives played a significant role in the women's ability to access and use them. As such, this underscores the need for interventional policies that prioritized the male as much as women, while also emphasizing the benefits of contraceptive use to military wives and men who could be apathetic toward contraceptive use due to the prolonged absence of military men from the home.

## Data Availability

The raw data supporting the conclusions of this article will be made available by the authors, without undue reservation.
