# Supplementary material for: The relevance of knowledge, perception, and factors that influence contraceptive use among married women living in Uaddara Barracks, Ghana
Source: Front Glob Womens Health. 2023 Aug 15;4:1110024. doi: 10.3389/fgwh.2023.1110024 (PMC10465326; doi:10.3389/fgwh.2023.1110024)
Supplement: Supplementary file 1 [file Datasheet1.pdf]

The Relevance of Knowledge, Perception, and Factors that influence Contraceptive Use Among Married Women Living in Uaddara Barracks, Ghana.

Daisy Afra Lumor[1*], Christian Obirikorang[2], Emmanuel Acheampong[2,3,4], Yaa Obirikorang[5], Hubert Owusu[2], Sam Newton[6]

[1] Department of Midwifery, St. Patrick's Nursing and Midwifery Training School, Offinso, Ghana
[2] Department of Molecular Medicine, School of Medicine and Dentistry, Kwame Nkrumah University of Science and Technology, Kumasi, Ghana
[3] Department of Genetic and Genome Biology, University of Leicester, Leicester United Kingdom
[4] Centre for Precision Health, School of Medical and Health Sciences, Edith Cowan University, Joondalup, WA, Australia
[5] Department of Nursing, Garden City University College, Kumasi, Ghana
[6] Department of Global and International Health, School of Public Health, Kwame Nkrumah University of Science and Technology, Kumasi, Ghana

Table S1: Contraceptive use among participants

Statement	n (%)
**What type of Contraceptive have you ever used? (N=286)**	
Injection	96(33.6)
Pills	114(39.8)
Condoms	89(31.1)
Periodic abstinence/ fertility awareness	31(10.8)
Intrauterine Device (IUD)	4(1.4)
Implants	4(1.4)
Withdrawal	31(10.8)
Spermicide	2(0.7)
Tubal ligation	2(0.7)
**What type of contraception method are you currently using? (N=180)**	
Injection	48 (26.7)
Pills	44 (24.4)
Condoms	36 (20.0)
Periodic abstinence/ fertility awareness	37 (20.6)
Intrauterine Device (IUD)	14 (7.8)
Implants	6 (3.3)
Withdrawal	15 (8.3)
Tubal ligation	2(1.1)
**If currently using contraceptives, was it your opinion? (N= 186)**	
Yes	150 (80.6)
**Experience of side effects of family planning method after application**	
Never	180 (51.4)
Seldom	102 (29.2)
Often	
**Is your husband in support of contraceptive use?**	
Yes	281 (80.3)
**Has your husband used the male condom?**	
Yes	235 (67.9)

Table S2: Awareness and knowledge of contraceptive use among the study participants

Statement	n (%)
**Have you ever heard of contraceptives?**	
Yes	341(97.4)
**How did you hear about contraceptive methods? (N=341)**	
From friends/relatives	209(61.3)
From the media/internet	220(64.5)
From a doctor	115(33.7)
From a midwife	190(55.7)
From health talk programs	66(19.4)
**What modern contraceptive method do you know? (N=341)**	
Injection	241(70.7)
Pills	186(54.5)
Condoms	254(74.5)
Sterilization	112(32.8)
Intrauterine Device (IUD)	152(44.6)
Implants	146(42.8)
Tubal ligation	113(33.1)
Diaphragm	77(22.6)
**What traditional contraceptive method do you know? (N=341)**	
None	18(5.3)
Withdrawal	179(52.5)
Periodic abstinence	44(12.9)
Lactational Amenorrhea Method	31(9.1)
Two methods only	35(10.3)
All methods	34(10.0)
**Do you know the source of contraceptives?**	
Yes	220(64.5)
**If yes, where? (N=220)**	
Pharmacy	97(44.0)
Community Based Outreaches	16(7.3)
Midwife	21(9.5)
Family Planning Unit	124(56.4)

Table S3: Study participants'' perception about contraceptive use

Statement	Don't know n (%)	Agree n (%)	Disagree n (%)
**1. Modern contraceptives services and commodities are inaccessible**	50 (14.3)	56 (16.0)	244 (69.7)
**2. It's not easy to discuss sexual issues with partner**	19 (5.4)	114 (32.6)	217 (62.0)
**3. Couple counseling can improve male involvement in contraceptive use**	21 (6.0)	248 (70.9)	81 (23.1)
**4. Contraceptives are for females only**	6 (1.7)	42 (12.0)	302 (86.3)
**5. Contraceptives are acceptable in the barracks community**	75 (21.4)	223 (63.7)	52 (14.9)
6. **Contraceptives benefit males too**	20 (5.7)	293 (83.7)	37 (10.6)
**7. Contraceptives is not for the poor**	17 (4.9)	154 (44.0)	179 (51.1)
**8. It is wrong to use contraceptives**	30 (8.6)	30 (8.6)	290 (82.9)

Table S4: Married life of study participants

Variable	Frequency (n=350)	Percentage (%)
**Length of married life (years)**		
1-5	141	40.3
6-10	120	34.3
11-15	58	16.6
>15	31	8.9
**Number of children**		
None	20	5.7
1-2	159	45.4
3-4	132	37.7
5-6	33	9.4
>6	6	1.7
**Number of children alive**		
Not applicable	20	5.7
1-2	159	45.4
3-4	143	40.9
5-6	21	6.0
7-8	6	1.7
**Number of children wanted**		
1-2	63	18.0
3-4	242	69.1
5-6	27	7.7
7-8	18	5.1
**Desire for a male child**		
No	71	20.3
Yes	167	47.7
Don’t mind gender	112	32.0
**Desire for a female child**		
No	95	27.1
Yes	143	40.9
Don’t mind gender	112	32.0
**Problems in taken decisions**		
No	214	61.1
Yes	136	38.9
